# Solution of fractional bioheat equation in terms of Fox’s H-function

**DOI:** 10.1186/s40064-016-1743-2

**Published:** 2016-02-03

**Authors:** R. S. Damor, Sushil Kumar, A. K. Shukla

**Affiliations:** Department of Applied Mathematics and Humanities, S. V. National Institute of Technology, Surat, 395 007 India

**Keywords:** Fractional bioheat equation, Caputo derivative, Riesz–Feller derivative, Fox’s H-function, 26A33, 35R11, 80A20

## Abstract

Present paper deals with the solution of time and space fractional Pennes bioheat equation. We consider time fractional derivative and space fractional derivative in the form of Caputo fractional derivative of order $\alpha \in \left( 0,1\right]$ and Riesz–Feller fractional derivative of order $\beta \in \left( 1,2\right]$ respectively. We obtain solution in terms of Fox’s H-function with some special cases, by using Fourier–Laplace transforms.

## Background

The transfer of heat in skin tissue is mainly a heat conduction process, which is coupled to several additional complicated physiological process, including blood circulation, sweating, metabolic heat generation and sometimes heat dissipation via hair or fur above the skin surface (Ozisik 1985). Accurate description of the thermal interaction between vasculature and tissue is essential for the advancement of medical technology in treating fatal disease such as tumors and skin cancer. Mathematical model has been used significantly in the analysis of hyperthermia in treating tumors, cryosurgery, fatal-placental studies, and many other applications (Minkowycz et al. 2009).

Fractals and fractional calculus have been used to improve the modelling accuracy of many phenomena in natural science. The most important advantage of using fractional calculus approach is due to its non-local property. This means that the next state of a system depends not only upon its current state but also upon all of its historical states. Many researchers worked on fractional partial differential equations and gave very important results. Mainardi et al. (2005) obtained the fundamental solution of Cauchy problem for the space–time fractional diffusion equation in terms of H-function, Langlands (2006) gave the solution of a modified fractional diffusion equation on an infinite domain, Salim and El-Kahlout (2009) discussed exact solution of time fractional advection dispersion equation with reaction term, Saxena et al. (2006) obtained solution of generalized fractional kinetic equation in terms of Mittag-Leffler function, Haubold et al. (2011a) obtained solution of a fractional reaction diffusion equation in closed form, Huang and Guo (2010) gave the fundamental solutions to a class of the time fractional partial differential equation for Cauchy problem in a whole-space domain and signalling problem in a half-space domain. Shang (2015) gave analytic solution of viral infection dynamics in vivo through a time-inhomogeneous Markov chain characterization by Lie algebraic approach.

In present study, we consider fractional form of Pennes bioheat equation by replacing first order time derivative by Caputo fractional derivative of order $\alpha \in (0,1]$ and second order space derivative by Riesz–Feller fractional derivative of order $\beta \in\left( 1,2\right]$ respectively. We make an attempt to solve the fractional model by dividing it into two sections. In section one, time fractional derivative is considered while in section two, space fractional derivative is taken into account. We apply Laplace–Fourier transform and obtain the solution in term of Fox H-function.

## Preliminaries and notations

Fractional derivative of order *α* is denoted as $_{a}D_{t}^{\alpha }f(t)$, the subscripts *a* and *t* denote the two limits related to the operation of fractional differentiation, which are called the terminal of fractional differentiation. If $\alpha$ is negative then it denotes the fractional integrals of arbitrary order.

### **Definition 1**

(*Kilbas et al.*^2006^) The Riemann–Liouville fractional derivative of order $\alpha > 0$ for $Real(\alpha )> 0$ and $m \in N, t > a$ is defined as

1 $$\begin{aligned} {a}D_{t}^{\alpha }f(t)=\left\{ \begin{array}{lcl} \frac{d^{m}}{dt^{m}}\left[ \frac{1}{\Gamma (m-\alpha )}\int _{a}^{t}\frac{f(\tau )}{(t-\tau )^{\alpha +1-m}} d\tau \right] ,&{}\quad \text{ for }&{} m-1< \alpha <m \\ \frac{d^{m}(f(t))}{dt^{m}}, &{}\quad \text{ for }&{}\alpha =m.\ \end{array}\right. \end{aligned}$$

### **Definition 2**

(*Kilbas et al.*^2006^) The Caputo fractional derivative of order $\alpha >0$, for $Real(\alpha )>0$ and $m\in N, t>a$ is defined as

2 $$\begin{aligned} _{a}^{c}D_{t}^{\alpha }(f(t))=\left\{ \begin{array}{lll} \left[ \frac{1}{\Gamma (m-\alpha )}\int _{a}^{t}\frac{f^{m}(\tau )}{(t-\tau )^{\alpha +1-m}}d\tau \right] ,&{} \quad \text{ for }&{} m-1< \alpha <m\\ \frac{d^{m}(f(t))}{dt^{m}}, &{}\quad \text{ for }&{}\alpha =m. \end{array}\right. \end{aligned}$$

### **Definition 3**

(*Kilbas et al.*^2010^) The Laplace transform of function *f*(*t*) denoted by *F*(*s*), *s* being the complex variable is defined as

3 $$\begin{aligned} F(s)=\overline{f}(s)=L\{{f(t);s}\}=\int _{0}^{\infty }e^{-st}f(t) dt; \end{aligned}\ \ \ t\in R^+$$

Inverse Laplace transform of *F*(*s*) is defined as

4 $$\begin{aligned} f(t)=L^{-1}\{{F(s);t}\}= \frac{1}{2\pi i} \int _{\gamma -i\infty }^{\gamma +i\infty }e^{st}F(s) ds; \end{aligned}\ \ \ t \in R^+, \gamma =R(s).$$

Laplace transform of Caputo derivative (Podlubny 1999) is given as

5 $$\begin{aligned} L\left[ _{0}^{c}D^{\alpha }_{t}f(t)\right] = s^{\alpha }F(s)-\sum _{k=0}^{n-1}s^{\alpha -k-1}f^{k}(0),\quad n-1<\alpha \le n. \end{aligned}$$

### **Definition 4**

(*Debnath and Bhatta*^2007^) The Fourier transform of a function *f*(*x*) is denoted by $F^{*}(k)$ and defined as

6 $$\begin{aligned} \textit{F}({f(x)})=F^{*}(k)=\int _{-\infty }^{\infty }e^{-ik x}f(x)dx, \end{aligned}$$

where, *f*(*x*) is continuous and absolutely integrable in $(-\infty ,\infty ).$ Inverse Fourier transform is defined as

7 $$\begin{aligned} F^{-1}(F^{*}(k))=\frac{1}{2\pi }\int _{-\infty }^{\infty }e^{ikx}F^{*}(k)dk. \end{aligned}$$

Fourier cosine transform and Inverse Fourier cosine transform are defined as (Debnath and Bhatta 2007)

8 $$\begin{aligned} F_{c}(f(x)) &= {} F_{c}^{*}(k)=\int _{0}^{\infty }\cos ({kx})f(x)dx, \end{aligned}$$

9 $$\begin{aligned} F_{c}^{-1}(F_{c}^{*}(k)) &= {} f(x)=\frac{2}{\pi }\int _{0}^{\infty }\cos (kx)F_{c}(k) dx. \end{aligned}$$

The Fourier convolution of two functions is defined (Debnath and Bhatta 2007) as

10 $$\begin{aligned} h*\phi =(h*\phi )(x)=\frac{1}{2\pi }\int _{-\infty }^{\infty }h{(x-t)}\phi (t)dt. \end{aligned}$$

### **Definition 5**

(*Kilbas et al.*^2010^) The Mellin transform of a function $\phi (t)$ is defined as

11 $$\begin{aligned} (\textit{M}\phi )(p)=\textit{M}[\phi (t)](p) =\phi ^{*}(s)=\int _{0}^{\infty }t^{s-1}\phi (t)dt; \end{aligned} \ \ \ t \in R^+=\left (0,\infty \right), \ \ s\in C.$$

and inverse Mellin transform as

12 $$\begin{aligned} (\textit{M}^{-1}g)(x)=\textit{M}^{-1}[g(s)](x) =\frac{1}{2\pi i}\int _{\gamma -i\infty }^{\gamma + i\infty }x^{-s}g(s)ds; \end{aligned} \ \ x \in R^+, \ \ \gamma=R(s).$$

### **Definition 6**

(*Kilbas et al.*^2006^) Riesz–Feller partial fractional derivative $\left( {D_{\theta ;x}^\beta u} \right) \left( {x,t} \right)$ defined, for $0<\beta \le 2$ and $\left| \theta \right| \le \min \left[ {\beta ,2 - \beta } \right]$ via the Fourier transform, by

13 $$\begin{aligned} \left( {{F_x}\,D_{\theta ;x}^\beta u} \right) \left( {\sigma ,t} \right) = {\left| \sigma \right| ^\beta }{e^{i\left( {sign\left( \sigma \right) {{\theta \pi } \over 2}} \right) }}\left( {{F_x}\,u} \right) \left( {\sigma ,t} \right) ; \quad {\sigma \in R;\,t > 0} . \end{aligned}$$

### **Definition 7**

(*Podlubny*^1999^) Mittag-Leffler function for one parameter is denoted by $E_{\alpha }(z)$ and defined as

14 $$\begin{aligned} E_{\alpha }(z)=\sum _{k=0}^{\infty }\frac{z^{k}}{\Gamma {(\alpha k +1)}},\quad \alpha >0. \end{aligned}$$

Mittag-Leffler function for two parameter is denoted by $E_{\alpha },_{\beta }(z)$ and defined as

15 $$\begin{aligned} E_{\alpha },_{\beta }(z)=\sum _{k=0}^{\infty }\frac{z^{k}}{\Gamma {(\alpha k +\beta )}},\quad \alpha >0, \beta >0 . \end{aligned}$$

Podlubny (1999) reported the Laplace transform of a derivative of Mittag Leffler function as

16 $$\begin{aligned} L(t^{\alpha n +\beta -1}E^{n}_{\alpha },_{\beta }(\pm a t^{\alpha }))=\frac{s^{\alpha -\beta }\Gamma {(n+1)}}{(s^{\alpha }\mp {a})^{n+1}}, \end{aligned}$$

and inverse Laplace transform of (16) is also existing as

17 $$\begin{aligned} L^{-1}\left( \frac{s^{\alpha -\gamma }}{(s^{\alpha }\mp {a})^{n+1}} \right) =\frac{1}{\Gamma {(n+1)}}t^{\alpha n +\gamma -1}E^{n}_{\alpha },_{\gamma }(\pm a t^{\alpha }). \end{aligned}$$

### **Definition 8**

(*Mathai et al.*^2010^) The H- function is defined by means of a Mellin–Barners type integral in the following manner

$$\begin{aligned} H(z)& = H_{p,q}^{m,n}\left( z\left| _{(b_{q},B_{q})}^{(a_{p},A_{p})}\right. \right) =H_{p,q}^{m,n}\left( z\left| _{(b_{1},B_{1}),\ldots , (b_{q},B_{q})}^{(a_{1},A_{1}),\ldots ,(a_{p},A_{p})}\right. \right) \\& = \frac{1}{2\pi {\mathbf{{i}}}} \int _L \Theta (s) z^{-s} ds , \end{aligned}$$

where,

18 $$\begin{aligned} {\mathbf{{i}}} &= {} \sqrt{-1},\quad z\ne 0, \quad z^{-s}= exp(-s~{\log |z|+ {\mathbf{{i}}}~ arg{z}}), \nonumber \\ \Theta (s &)= {} \frac{\prod _{j=1}^{m}\Gamma (b_{j}+B_{j} {s}){\prod _{j=1}^{n}\Gamma (1-a_{j}-A_{j} {s})}}{\prod _{j=m+1}^{q}\Gamma (1-b_{j}-B_{j} {s}){\prod _{j=n+1}^{p}\Gamma (a_{j}+A_{j} {s})}}, \end{aligned}$$

and an empty product is interpreted as unity, $m,n,p,q\in N_{0}$ with $0 \le n \le p, 0 \le m \le q, A_{j},B_{j}\in R_{+}, a_{j},b_{j}\in C, j=1,\ldots ,p;\quad j=1,\ldots ,q,$ such that $A_{{j}}(b_{l}+k)\ne B_{l}(a_{j}-\lambda -1), k,\lambda \in N_{0};\quad {j}{=1,\ldots ,n;}\quad {j=1,\ldots ,m.}$ where $N_{0}= 0,1,2,\ldots ,R=(-\infty ,\infty ),~~R_{+}=(0,\infty )$ and *C* being the complex number field. The counter $\Omega$ is infinite contour which separates all the poles of $\Gamma (1-a_{{j}} +sA_{l}),~~ {j}=1,\ldots ,n.$

The relation between derivative of Mittag-Leffler function and H-Function (Langlands 2006) is given as

19 $$\begin{aligned} E^{m}_{\alpha ,\beta }(\eta )=H^{1,1}_{1,2}\left[ - \eta \vert ^{(-m,1)}_{(0,1),(1-\alpha m-\beta , \alpha )} \right] . \end{aligned}$$

## Fractional bioheat equation

The Pennes bioheat model (Pennes 1948) is widely used for study of the heat transfer in skin tissue due to its simplicity, ease application and effectiveness. Pennes (1948) suggested that the rate of heat transfer between blood and tissue is proportional to the product of the volumetric perfusion rate and difference between the arterial blood temperature and the local tissue temperature. Pennes equation is employed to describe the heat transfer process as

20 $$\begin{aligned} \rho c\frac{\partial T}{\partial t}= k \frac{\partial ^{2} T}{\partial x^2}+ W_{b}c_{b}(T_{a}-T) + Q_{met}+Q_{ext}, \end{aligned}$$

where $\rho$, *c* and *k* represent density, specific heat and thermal conductivity respectively. *T*, *t* and *x* represent, temperature, time and distance respectively; the subscript *b* denotes for blood. $T_{a}$ and $W_{b}$ are artillery temperature and blood perfusion rate respectively. $Q_{met}$ and $Q_{ext}$ are metabolic heat generation and external heat source in skin tissue respectively.

Considering Eq. (20) and replacing first order time derivative by Caputo fractional derivative of order $\alpha \in (0,1]$ and second order space derivative by Riesz–Feller fractional derivative of order $\beta \in \left( 1,2\right]$. The fractional form of Pennes bioheat equation is given as

21 $$\begin{aligned} \rho c \frac{\partial ^\alpha T}{\partial t^\alpha }= k \frac{\partial ^\beta T}{\partial x^\beta }+ W_{b}c_{b}(T_{a}-T) + Q_{met}. \end{aligned}$$

Initial and boundary conditions of Eq. (21) are given below as

22 $$\begin{aligned}&T(x,0)= T_{a}, \end{aligned}$$

23 $$\begin{aligned}&T(x,t)\vert _{x\rightarrow \pm \infty }= 0. \end{aligned}$$

To find solution of Eq. (21) to (23). We divide it into two parts, in first part, the time fractional is considered while in second part, the space fractional is considered.

## Time fractional bioheat equation

On setting $\beta =2$ in Eq. (21), this reduces to the following equation

24 $$\begin{aligned} \rho c \frac{\partial ^\alpha T}{\partial t^\alpha }= k \frac{\partial ^{2} T}{\partial x^{2}}+ W_{b}c_{b}(T_{a}-T) + Q_{met}.\end{aligned}$$

For converting Eq. (24) in dimensionless variable, we consider

25 $$\begin{aligned} \zeta =\left( \frac{W_{b}c_{b}}{k}\right) ^\frac{1}{2}~x, \quad \eta =\left( \frac{W_{b}c_{b}}{\rho c}\right) ^\frac{1}{\alpha }~t, \quad \theta = \frac{T-T_{a}}{T_{0}}, \quad \phi =\frac{Q_{met}}{T_{0}W_{b}c_{b}}. \end{aligned}$$

On making the use of dimensionless variables as Eq. (25). We can reduce Eqs. (22) to (24) into Eq. (26) to (28) as

26 $$\begin{aligned} \frac{\partial ^{\alpha }\theta }{\partial \eta ^{\alpha }}+ \theta= & {} \frac{\partial ^{2}\theta }{\partial \zeta ^{2}} +\phi , \end{aligned}$$

27 $$\begin{aligned} \theta (\zeta ,0)= & {} 0, \end{aligned}$$

28 $$\begin{aligned} \theta (\zeta ,\eta )\vert _{\zeta \rightarrow \pm \infty }= & {} 0. \end{aligned}$$

### Solution

On replacing fractional derivative by Caputo fractional derivative and taking the Fourier–Laplace transform (Haubold et al. 2011b) of Eq. (26) and further using Eq. (5) and Eq. (6), we get

$$\begin{aligned} s^{\alpha }\overline{\theta }^*(\omega ,s)-\theta (\zeta ,0) +\overline{\theta }^*(\omega ,s)=-\omega ^{2}\overline{\theta }^*(\omega ,s) +\phi 2\pi \delta (\omega )\frac{1}{s}~, \end{aligned}$$

where $\overline{\theta }^*$ is Fourier Laplace transform of $\theta$, on further simplifications, we get

29 $$\begin{aligned} \overline{\theta }^*(\omega ,s)= 2\pi \phi \delta (\omega )\frac{1}{1+\omega ^{2}} \left( \frac{1}{s}\right) -2\pi \phi \delta (\omega ) \frac{1}{1+\omega ^{2}}\sum _{m=0}^{\infty }(-1)^{m} \frac{s^{\alpha -1}}{(s^{\alpha }+\omega ^{2})^{m+1}}. \end{aligned}$$

On taking inverse Laplace transform of Eq. (29) and using Eq. (17), we get

30 $$\begin{aligned} \theta ^*(\omega ,\eta )= 2\pi \phi \delta (\omega )\frac{1}{1+\omega ^{2}}-2\pi \phi \delta (\omega )\frac{1}{1+\omega ^{2}}\sum _{m=0}^{\infty }(-1)^{m}\frac{\eta ^{\alpha m}}{m!}E^{m}_{\alpha }(-\omega ^{2} \eta ^{\alpha }). \end{aligned}$$

On replacing derivative of Mittag-Leffler function by H-function using Eq. (19), Eq. (30) reduces to the following form

31 $$\begin{aligned} \theta ^*(\omega ,\eta )& = 2\pi \phi \delta (\omega )\frac{1}{1+\omega ^{2}}\nonumber \\& \quad -\,2\pi \phi \delta (\omega )\frac{1}{1+\omega ^{2}}\sum _{m=0}^{\infty }(-1)^{m}\frac{\eta ^{\alpha m}}{m!}H^{1,1}_{1,2}\left[ \omega ^{2}\eta ^{\alpha }\vert ^{(-m,1)}_{(0,1),(-\alpha m, \alpha )} \right]. \end{aligned}$$

Now we consider

32 $$\begin{aligned} G_{m}^*(\omega ,\eta )=H^{1,1}_{1,2}\left[ \omega ^{2}\eta ^{\alpha }\vert ^{(-m,1)}_{(0,1),(-\alpha m, \alpha )} \right] , \end{aligned}$$

to invert Fourier transform of Eq. (31), we first invert Fourier transform of Eq. (32), by taking its Mellin transform, since Mellin transform of H-function (Srivastava et al. 1982) is given below as

33 $$\begin{aligned} \textit{M}\left\{ H^{m,n}_{p,q}\left[ ax \vert ^{(a_{p},A_{p})}_{(b_{q},B_{q})} \right] \right\} (z)=a^{-z}\frac{\left\{ \prod ^{m}_{j=1} \Gamma (b_{j}+sB_{j}) \right\} }{\left\{ \prod ^{q}_{j=m+1} \Gamma (1-b_{j}-sB_{j}) \right\} }\frac{\left\{ \prod ^{n}_{j=1} \Gamma (1-a_{j}-sA_{j}) \right\} }{\left\{ \prod ^{p}_{j=n+1} \Gamma (a_{j}+sA_{j}) \right\} },\nonumber \\ \end{aligned}$$

with the following conditions

$$\begin{aligned} \delta= & {} \sum _{j=1}^{q}\beta _{j}- \sum _{j=1}^{p}\alpha _{j} > 0,\\ A= & {} \sum _{j=1}^{n}\alpha _{j}- \sum _{j=n+1}^{p}\alpha _{j}+\sum _{j=1}^{m}\beta _{j}- \sum _{j=m+1}^{q}\beta _{j} > 0,\quad |arg(a)|<\frac{1}{2} A \pi , \end{aligned}$$

and

$$\begin{aligned} -min _{1\le j \le m}\left[ R\left( \frac{b_{j}}{\beta _{j}}\right) \right] < R(z)< min _{1\le j \le n}\left[ R\left( \frac{1-a_{j}}{\alpha _{j}}\right) \right] ~. \end{aligned}$$

Further Mellin transform of a Fourier transform is given as (Langlands 2006)

34 $$\begin{aligned} \textit{M} \lbrace F[\psi (x)](q)\rbrace {(z)}= 2 \Gamma (z)cos\left( \frac{\pi z}{2}\right) \,M\lbrace \psi (x)\rbrace (1-z), \end{aligned}$$

therefore the Mellin transform of Eq. (32) is

35 $$\begin{aligned} \textit{M}\left( G_{m}(\zeta ,\eta )\right) (z)=\left( \frac{1}{2}\right) \eta ^{-\frac{\alpha }{2}}\frac{\Gamma (z)\Gamma \left( \frac{1}{2} +m-\frac{z}{2}\right) \Gamma \left( \frac{1}{2}+\frac{z}{2}\right) }{\Gamma \left( 1-\frac{\alpha }{2}+ \frac{\alpha z}{2}\right) \Gamma \left( \frac{1}{2}-\frac{z}{2}\right) }. \end{aligned}$$

On taking the inverse Fourier transform of Eq. (31) by using Eq. (10), we get

36 $$\begin{aligned} \theta (\zeta ,\eta )& = \phi \exp ({-\vert \zeta \vert })\nonumber \\&-\,\phi \sum _{m=0}^{\infty }(-1)^{m}\frac{\eta ^{\alpha m}}{m!}\int _{-\infty }^{\infty }\exp ({-\vert \zeta \vert })\frac{1}{2\eta ^{\alpha /2}}H^{2,1}_{2,3}\left[ \frac{\vert \zeta -\tau \vert }{\eta ^{\alpha /2}}\vert ^{(\frac{1}{2}-m,\frac{1}{2}),(1-\frac{\alpha }{2},\frac{\alpha }{2})}_{(0,\frac{1}{2}),(\frac{1}{2},\frac{1}{2}),(\frac{1}{2},\frac{1}{2})} \right] d\tau .\nonumber \\ \end{aligned}$$

### Special case

On considering time fractional bioheat equation (24) in semi infinite space with $Q_{met}=0(\phi =0)$ and Neumann boundary condition on the left hand boundary, i.e. Eq. (23) is replaced by the following equation

37 $$\begin{aligned} -k\frac{\partial T}{\partial x}|_{x=0}= q_{0},\quad T(x,t)\vert _{x\rightarrow \infty }= 0, \end{aligned}$$

for dimensionless variable, we consider

38 $$\begin{aligned} \zeta =\left( \frac{W_{b}c_{b}}{k}\right) ^\frac{1}{2}~x, \quad \eta =\left( \frac{W_{b}c_{b}}{\rho c}\right) ^\frac{1}{\alpha }~t, \quad \theta = \frac{T-T_{a}}{q_{0}}(k W_{b}c_{b})^\frac{1}{2}. \end{aligned}$$

On making the use of Eq. (38), then Eqs. (24) and (37) reduce to

39 $$\begin{aligned}&\frac{\partial ^{\alpha }\theta }{\partial \eta ^{\alpha }}+ \theta =\frac{\partial ^{2}\theta }{d\zeta ^{2}}, \end{aligned}$$

40 $$\begin{aligned}&\frac{\partial \theta }{\partial \zeta }\vert _{\zeta =0}=-1\quad \theta (\zeta ,\eta )\vert _{\zeta \rightarrow \infty }=0. \end{aligned}$$

Further, on applying the Fourier cosine–Laplace transform to Eq. (39) and using Eq. (5) and Eq. (8), we obtain

$$\begin{aligned} s^{\alpha }\overline{\theta }^*(\omega ,s)-\theta (\zeta ,0) +\overline{\theta }^*(\omega ,s)=-\omega ^{2}\overline{\theta }^*(\omega ,s)+1~, \end{aligned}$$

where,

$$\begin{aligned} \overline{\theta }^*(\omega ,s)= F_{c}L\left\{ \theta (\zeta ,\eta )\right\} =\int _{0}^{\infty }\cos (\omega \zeta )\int _{0}^{\infty }e^{-s\eta }\theta (\zeta ,\eta )d{\zeta }d{\eta }~, \end{aligned}$$

further simplification leads us

41 $$\begin{aligned} \overline{\theta }^*(\omega ,s)=\sum _{m=0}^{\infty }(-1)^{m} \frac{1}{(s^{\alpha }+\omega ^{2})^{m+1}}. \end{aligned}$$

Now taking inverse Laplace transformation to Eq. (41), as in light of Eq. (17), we get

42 $$\begin{aligned} \theta ^*(\omega ,s)=\sum _{m=0}^{\infty }(-1)^{m}\frac{1}{\Gamma {(m+1)}} \eta ^{\alpha m +\alpha -1}E^{m}_{\alpha ,{\alpha }}(- \omega ^2 \eta ^{\alpha }). \end{aligned}$$

Afterwards using Eq. (19), then Eq. (42) reduces to the following form

43 $$\begin{aligned} \theta ^*(\omega ,s)=\sum _{m=0}^{\infty }(-1)^{m}\frac{1}{\Gamma {(m+1)}}\eta ^{\alpha m +\alpha -1}H^{1,1}_{1,2}\left[ -\omega ^2 \eta ^\alpha \vert ^{(-m,1)}_{(0,1),(1-\alpha m-\alpha , \alpha )} \right]. \end{aligned}$$

Now we find it’s inverse Fourier cosine transform by using Eq. (33) and the following result (Langlands 2006) as

44 $$\begin{aligned} \frac{\sqrt{\pi }}{2}{} \textit{M} \lbrace F_{c}[\psi (x)](q)\rbrace {(z)}= \Gamma (z)\cos \left( \frac{\pi z}{2}\right) \textit{ M}\lbrace \psi (x)\rbrace (1-z). \end{aligned}$$

Thus we get

45 $$\begin{aligned} \theta (\zeta ,\eta )=\sum _{m=0}^{\infty }(-1)^{m}\frac{1}{\Gamma {(m+1)}}\eta ^{\alpha n +\alpha -1}\frac{1}{(4\pi \eta ^\alpha )^{1/2}}H^{2,1}_{2,3}\left[ -\frac{\vert \zeta \vert }{\eta ^\alpha /2} \vert ^{(\frac{1}{2}-m,\frac{1}{2}),(1-\alpha /2,\alpha /2)}_{(0,\frac{1}{2}),(\frac{1}{2},\frac{1}{2}),(\frac{1}{2},\frac{1}{2})} \right] .\nonumber \\ \end{aligned}$$

This is the solution of special case for the time fractional bioheat equation in the form of well knows H-function (see in details Mathai et al. 2010 ).

## Space fractional bioheat equation

we consider $\alpha =1$ in Eq. (21) and dimensionless variables as

46 $$\begin{aligned} \zeta =\left( \frac{W_{b}c_{b}}{k}\right) ^\frac{1}{\beta }~x, \quad \eta =\left( \frac{W_{b}c_{b}}{\rho c}\right) ~t, \quad \theta = \frac{T-T_{a}}{T_{0}}, \quad \phi =\frac{Q_{met}}{T_{0}W_{b}c_{b}}, \end{aligned}$$

since Riez–Feller derivative of constant is not zero, therefore a new space dependent term $f(\zeta )$ is introduced for simplification.

47 $$\begin{aligned} \frac{\partial \theta }{\partial \eta } + \theta =\frac{\partial ^{\beta }\theta }{\partial \zeta ^{\beta }}+f(\zeta ), \end{aligned}$$

where $f(\zeta )=\frac{W_{b}c_{b}T_{a}}{\Gamma (1-\beta )T_{0}}\zeta ^{-\beta }$, initial and boundary conditions are given in Eqs. (27) and  (28) respectively.

### Solution

On taking Fourier–Laplace transform of Eq. (47), and using Eq. (13), this leads us

$$\begin{aligned} s\overline{\theta }^*(\omega ,s)-\theta (\zeta ,0) +\overline{\theta }^*(\omega ,s)=-\vert \omega \vert ^{\beta } \overline{\theta }^*(\omega ,s)+f^{*}(\zeta )\frac{1}{s}~, \end{aligned}$$

further simplification gives,

48 $$\begin{aligned} \overline{\theta }^*(\omega ,s)= f^{*}(\zeta )\frac{1}{1+\vert \omega \vert ^{\beta }}\left( \frac{1}{s}-\frac{1}{(s+1+\vert \omega \vert ^{\beta })}\right). \end{aligned}$$

On taking inverse Laplace transform of Eq. (47) and using Eq. (17), this yields

49 $$\begin{aligned} \theta ^*(\omega ,\eta )= f^{*}(\zeta )\frac{1}{1+\vert \omega \vert ^{\beta }}-f^{*}(\zeta )E_{1,1}(-\eta )\frac{1}{1+\vert \omega \vert ^{\beta }}E_{1,1}(-\vert \omega \vert ^{\beta }\eta ), \end{aligned}$$

afterwards taking the inverse Fourier transform of Eq. (48) and employing the following results of Haubold et al. (2011b), which is given as

50 $$\begin{aligned} F^{-1}\left\{ E_{\beta ,\gamma }(-\eta t^{\beta }\vert k \vert ^{\alpha })\right\}& = \frac{1}{\alpha \vert x \vert }H^{2,1}_{3,3}\left[ \frac{\vert x \vert }{(\eta t^{\beta })^{1/\alpha }} \vert ^{(1,\frac{1}{\alpha })(\gamma ,\frac{\beta }{\alpha }),(1,\frac{1}{2})}_{(1,1/\alpha ),(1,1),(1,\frac{1}{2})} \right] ,\nonumber \\ F^{-1}\left\{ E_{1,1}(-\eta \vert \omega \vert ^{\beta })\right\}& = \frac{1}{\beta \vert \zeta \vert }H^{2,1}_{3,3}\left[ \frac{\vert \zeta \vert }{(\eta ^{1/\beta })} \vert ^{(1,\frac{1}{\beta })(1,\frac{1}{\beta }),(1,\frac{1}{2})}_{(1,1/\beta ),(1,1),(1,\frac{1}{2})} \right] ~. \end{aligned}$$

Finally, we arrive at

51 $$\begin{aligned} \theta (\omega ,\eta )& = \frac{1}{2\pi }\int _{-\infty }^{\infty }f(\zeta ) \exp (-\vert \zeta \vert ^{\frac{\beta }{2}})\nonumber \\&- \,E_{1,1}(-\eta )\frac{1}{2\pi }\int _{-\infty }^{\infty } f(\zeta )\exp (-\vert \zeta \vert ^{\frac{\beta }{2}})\frac{1}{\beta \vert \zeta \vert } H^{2,1}_{3,3}\left[ \frac{\vert \zeta -\tau \vert }{(\eta ^{1/\beta })} \vert ^{(1,\frac{1}{\beta })(1,\frac{1}{\beta }),(1,\frac{1}{2})}_{(1,1/\beta ),(1,1),(1,\frac{1}{2})} \right] d\tau .\nonumber \\ \end{aligned}$$

This is a solution of space fractional bioheat equation.

## Conclusion

In this paper, we have investigated the temperature distribution of the biological tissue based on fractional bioheat transfer model. The fractional bioheat transfer equation is solved using integral transforms which yields analytical solution. This analytical solution may be useful in measurement of thermal parameters, reconstruction of temperature field, thermal diagnosis and thermal treatments.
